# Isolated inferior vena cava aneurysm: a case report

**DOI:** 10.15171/jcvtr.2019.12

**Published:** 2019-03-14

**Authors:** Niki Tadayon, Seyyed Mohammadreza Kalantar-Motamedi, Sina Zarrintan, Ali Tayyebi

**Affiliations:** ^1^Division of Vascular and Endovascular Surgery, Department of General & Vascular Surgery, Shahid Beheshti University of Medical Sciences, Tehran, Iran; ^2^Phlebology Research Group, Shahid Beheshti University of Medical Sciences, Tehran, Iran; ^3^Shohada-Tajrish Hospital, Shahid Beheshti University of Medical Sciences, Tehran, Iran

**Keywords:** Aneurysm, Inferior Vena Cava, Venorrhaphy, Right Medial Visceral Rotation

## Abstract

We report a rare case of inferior vena cava (IVC) aneurysm in a 22-year old Afghan-Iranian male patient. CT scan illustrated a saccular aneurysm of IVC originating from right side of the IVC below the renal veins (a saccular type 3 IVC aneurysm). We planned open resection and repair of the aneurysm. The patient had well recovery after the operation and his follow-up did not reveal any morbidity. IVC aneurysm is a rare clinical entity. Its diagnosis necessitates precise clinic suspicion and the management is based on anatomical location and associated anomalies.

## Introduction

Inferior vena cava (IVC) aneurysm is a rare venous abnormality with potential clinical consequences.^1^ IVC aneurysm could have potential morbidity and mortality and its diagnosis and management is of clinical significance.^2^ We present a rare case of incidental IVC aneurysm which was diagnosed and managed successfully.

## Case Report

We report a rare case of IVC aneurysm in a 22-year old Afghan-Iranian male patient. The patient had a history of blunt abdominal trauma one week prior to his referral to the emergency department of our center. On his initial abdominal trauma, a complete physical examination and focused assessment with sonography for trauma (FAST) was done. The investigations were normal and the patient was discharged from the emergency department. The patient has had vague abdominal pain after his discharge.

**Figure 1:**
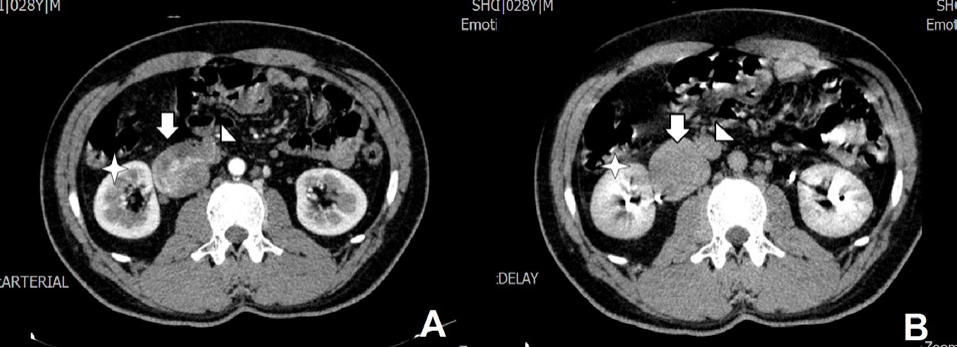
Contrast-enhanced CT abdominopelvic CT illustrating saccular type III IVC aneurysm; arterial phase (A) and delayed phase (B); the aneurysm (arrow); IVC (arrowhead); right kidney (strike)

**Figure 2:**
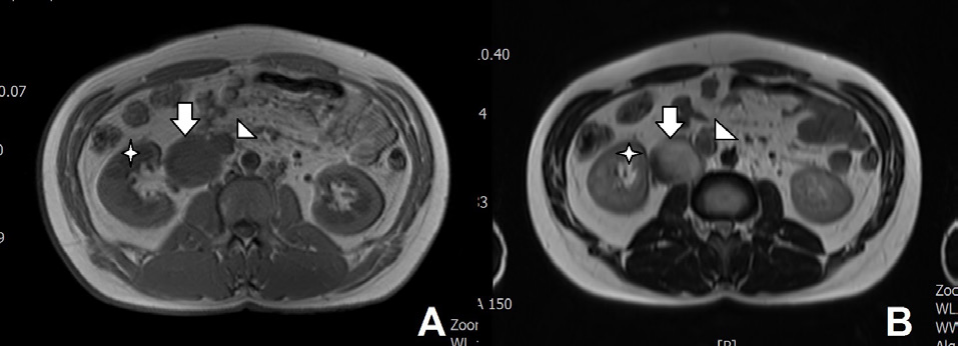
Magnetic resonance imaging confirming the diagnosis of IVC aneurysm; T1 (A) and T2 (B); the aneurysm (arrow); IVC (arrowhead); right kidney (strike).

On the referral of the patient to our center, we planned an abdominopelvic computed tomography (CT) scan with oral and IV contrast. The scan illustrated an IVC saccular aneurysm originating from right side of the IVC below the renal veins (Figure 1). We assumed two possible etiologies. The aneurysm could incidentally and in another hand it could be related to the patient’s recent history of abdominal trauma. Magnetic resonance venography was also conducted and it also confirmed the diagnosis of a saccular type III IVC aneurysm (Figure 2).

**Figure 3:**
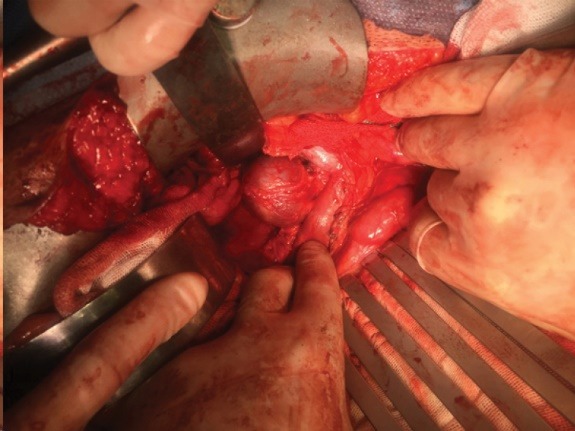
Intraoperative illustration of IVC aneurysm; the aneurysm was located below the renal veins.

**Figure 4:**
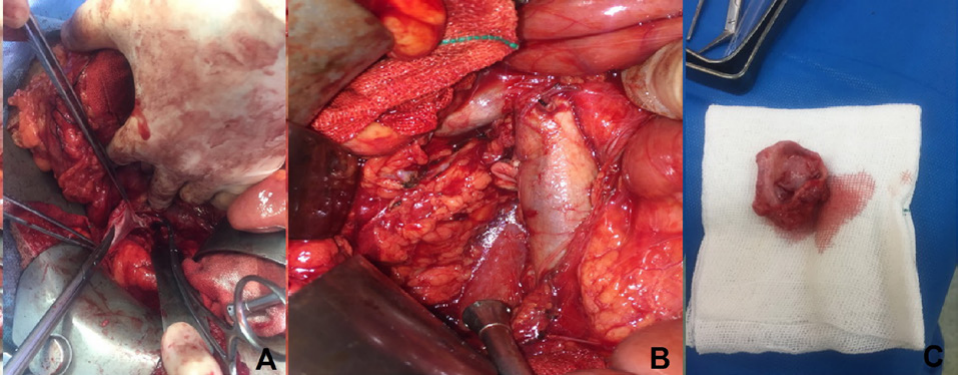
The aneurysm was resected by placement of a partial Satinsky clamp and venorrhaphy; resection (A); venorrhaphy of the neck of the aneurysm (B); the resected aneurysm (C)

We planned open resection and repair of the aneurysm. A midline laparotomy was done. After thorough exploration of the abdominal and pelvic cavities, a right medial visceral rotation was conducted by mobilization of the right colon and a Kocher maneuver (The Cattel-Braasch Maneuver). The right kidney was left in situ. The entire sub-hepatic IVC was exposed. A saccular aneurysm with dimensions of 4*5 cm was found on exploration (Figure 3). The aneurysm was located below the renal veins and the neck of the aneurysm was at the right side. The aneurysm was confined to the infrarenal IVC and there was not any associated venous anomaly. Thus, it was a type III saccular IVC aneurysm. A partial Satinsky clamp was applied posterior and left to the site of aneurysm origin on IVC and a longitudinal incision was done anterior to the neck of the aneurysm. Then, the entire aneurysm was resected. The neck of the aneurysm was closed with lateral venorrhaphy by running 6.0 polypropylene sutures (Figure 4).

**Figure 5:**
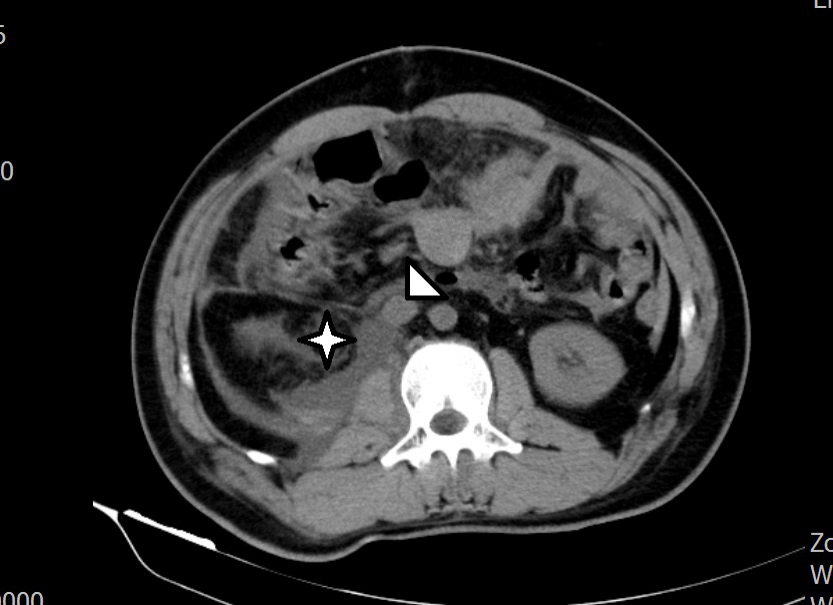
Postoperative CT scan illustrating normal appearance of IVC; IVC (arrowhead); right kidney (strike)

The patient had well recovery after the operation. Postoperative anticoagulation was administered by unfractionated heparin and warfarin. Warfarin anticoagulation was continued for three months to prevent venous thrombosis and probable pulmonary embolism. The patient’s follow-up did not reveal any morbidity. Postoperative CT scan was also conducted on seventh postoperative day. Postoperative appearance of IVC was normal (Figure 5).

## Discussion

IVC aneurysm is a rare clinical entity. Its diagnosis necessitates precise clinic suspicion and the management is based on anatomical location and associated anomalies. Due to the paucity of literature data regarding the history of IVC aneurysms, there is no consensus on their treatment.^1^ Pathophysiologic factors that cause IVC aneurysm include trauma, inflammation, uncontrolled hypertension, congenital anomalies.^2-4^ However, a number of IVC aneurysms have been shown to be idiopathic.^5,6^ We report a rare case of saccular type III IVC aneurysm without any associated anomaly of systemic disease in an otherwise healthy young man. Our case had a recent history of blunt abdominal trauma.

IVC aneurysm is classified into four types: type I, aneurysms of the suprahepatic IVC without venous obstruction; type II, aneurysms located on IVC above or below the hepatic veins; type III, aneurysms confined to the infrarenal IVC without associated venous anomaly; and type IV, miscellaneous.^7,8^ Our case was a rare case of IVC aneurysm located below the renal veins. The location of the aneurysm was confirmed by preoperative CT. It has been proposed that thrombosis due to flow dynamics resulting in venous stasis may be more prominent in IVC aneurysms types II to IV, making their presentations as deep vein thrombosis.^2^ However, our presented case did not show any clinical and radiological signs of deep vein thrombosis and we assumed that the aneurysm was incidental or caused by recent trauma.

Montero-Baker et al^9^ did a comprehensive review on the management of IVC aneurysms in 2015. According to their systematic literature review, there were 54 reported cases of IVC aneurysms in a span of 42 years. Of their reviewed cases, 23 cases had type I IVC aneurysm (42.6%), 8 cases had type II IVC aneurysm (14.8%), 21 cases had type III IVC aneurysm (38.9%) and 2 cases had type IV IVC aneurysm (3.7%). Thus, type III IVC aneurysm is the second most common type and its diagnosis and management is of potential clinical concern. Because of the possibility of DVT in types II to IV IVC aneurysms, this should be considered in mind in differential diagnosis of DVT.^2^ Surgical management is the treatment of choice for IVC aneurysms^2-5^; however, medical management could be considered in some cases especially in asymptomatic type I IVC aneurysms. Medical management include anticoagulation and IVC filter placement.^9^ In addition, an endovascular stent-graft could be an attractive alternative.^10^

IVC aneurysm treatment is generally recommended because of potential life-threatening morbidities such as thrombosis and pulmonary embolism.^11^ In addition to open surgical exploration and resection, endovascular attempts have been reported in the literature. Michel and Alomari reported a type III saccular aneurysm in a 2.5-year-old male child which was manage by endovascular intervention successfully. They used embolization with coils and an Amplatzer vascular plug device.^12^

Weber et al reported a similar case of infrarenal IVC aneurysm which was managed by aneurysm resection and IVC repair.^13^ Davidovic et al also reported a symptomatic patient with thrombosed IVC aneurysms which was managed surgically.^14^ In addition, Makaloski and Schmidli reported a 16-year old girl with abdominal pain which was diagnosed by a huge IVC aneurysm and managed by surgical resection.^15^ Elliot et al reported a case of IVC aneurysm in a 16-year old patient who presented with deep vein thrombosis of right lower extremity.^16^ Unzueta-Roch et al reported a case of IVC aneurysm mimicking right renal mass in a five-month old infant which was managed by surgery.^17^ Similar cases of surgical management of IVC aneurysms have been reported by Deshpande et al^18^ and Lochbuehler et al^19^ Tansel et al reported another case of IVC aneurysm in a 14-year old boy who was followed-up periodically without surgery.^20^

In conclusion, IVC aneurysm is an extremely rare entity. However, its diagnosis and management is of potential clinical concern due to its significant morbidity and mortality. IVC aneurysms could be complicated by thrombosis and pulmonary embolism and even death. Thus, open surgery or endovascular therapy should be considered in its management. In the present case, the history of recent trauma and creation of an IVC aneurysm had the potential of rupture and thus we decided to do surgical correction. In addition, risk of thrombosis and subsequent pulmonary embolism was another indication for surgical intervention.

## Ethical approval

Ethical approval is not necessary for retrospective studied and case presentation in out institutional policies. However, informed consent has been obtained from the patient to publish this material.

## Competing interests

None.
